# Epinephrine injection monotherapy shows similar hemostatic efficacy to epinephrine injection combined therapy in high-risk patients (Forrest Ib) with bleeding ulcers

**DOI:** 10.1007/s00464-023-10152-4

**Published:** 2023-06-19

**Authors:** Jingsong Wang, Shan He, Guanqun Shang, Nonghua Lv, Xu Shu, Zhenhua Zhu

**Affiliations:** 1 Department of Gastroenterology, Digestive Disease Hospital, The First Affiliated Hospital of Nanchang University, 17 Yongwaizheng Street, Nanchang, 330006 Jiangxi China; 2 Department of Gastroenterology, Gaoxin Branch, The First Affiliated Hospital of Nanchang University, Nanchang, 330096 Jiangxi China; 3 Queen Mary School, Nanchang University, Nanchang, 330001 Jiangxi China

**Keywords:** Epinephrine injection monotherapy, Combined therapy, Rebleeding, Peptic ulcer bleeding, Propensity score analysis

## Abstract

**Background:**

Whether combination therapy has higher hemostatic efficacy than epinephrine injection monotherapy in different Forrest classifications is not clear. This study aimed to compare hemostatic efficacy between epinephrine injection monotherapy (MT) and combination therapy (CT) based on different Forrest classifications.

**Methods:**

We retrospectively analyzed peptic ulcer bleeding (PUB) patients who underwent endoscopic epinephrine injections or epinephrine injections combined with a second therapy between March 2014 and June 2022 in our center, and the patients were divided into MT group or CT group. Subsequently, a propensity score matching analysis (PSM) was performed and rebleeding rates were calculated according to Forrest classifications via a stratified analysis.

**Results:**

Overall, 605 patients who met the inclusion criteria were included, and after PSM, 173 patients in each of the CT and MT groups were included. For PUB patients with nonbleeding visible vessels (FIIa), the rebleeding rates by Days 3, 7, 14, and 30 after PSM were 8.8%, 17.5%, 19.3%, and 19.3% in the MT group, respectively, and rates were 0%, 4.1%, 5.5%, and 5.5% in the CT group, respectively, with significant differences observed between the two groups by Days 3, 7, 14, and 30 (P = 0.015, P = 0.011, P = 0.014, and P = 0.014, respectively). However, for PUB patients with oozing bleeding (FIb), the rebleeding rates by Days 3, 7, 14, and 30 after PSM were 14.9%, 16.2%, 17.6%, and 17.6% in the MT group, respectively, and rates were 13.2%, 14.7%, 14.7%, and 16.2% in the CT group, respectively, with no significant differences observed between the two groups by Days 3, 7, 14, and 30 (P = 0.78, P = 0.804, P = 0.644 and P = 0.825).

**Conclusion:**

Combined therapy has higher hemostatic efficacy than epinephrine injection monotherapy for PUB patients with visible blood vessel (FIIa) ulcers. However, epinephrine injection monotherapy is equally as effective as combined therapy for PUB patients with oozing blood (FIb) ulcers.

Peptic ulcer bleeding (PUB) is one of the most common and severe complications of peptic ulcers, accounting for the majority of acute nonvariceal upper gastrointestinal bleeding incidences [1–4]. Although the incidence of PUB has decreased and a better prognosis has been achieved with the development of endoscopic hemostasis and proton pump inhibitors (PPIs), 10–15% of PUB patients still experience rebleeding within 30 days after endoscopic hemostasis [1–8]. Endoscopy is essential for the diagnosis, stratification, and management of patients with bleeding ulcers. Moreover, endoscopic findings can be classified according to the Forrest classification for guidance regarding the need for intervention and risk of rebleeding (Forrest Ia: spurting bleeding, Ib: oozing bleeding, IIa: visible vessel, IIb: an adherent clot, IIc: a flat pigmented spot, and Forrest III: a clean base ulcer) [3, 9]. A variety of modalities are currently available for endoscopic therapy of ulcer bleeding, including injections (diluted epinephrine, sclerosing agents, and cyanoacrylate), thermal coagulation (monopolar, bipolar, multipolar, or heater probe), mechanical (clips, band ligation, and over-the-scope), and topical therapy (topical hemostatic spray/powder) [7, 9]. Although many authoritative guidelines and articles recommend combination therapy using epinephrine injections plus a second hemostasis modality for high-risk bleeding ulcers (especially with active spurting, active oozing, or nonbleeding visible vessels), epinephrine injections still represent the most common method of emergency endoscopic hemostasis and are widely used in clinical practice, especially in nontertiary hospitals that do not have the conditions and technology for combined endoscopic treatment. Furthermore, these injections have the advantages of low costs, safety, easy operation, and low technical requirements [9–18].

A meta-analysis of seven studies without a second look plus retreatment demonstrated a significant benefit of adding the second modality for further bleeding, surgery, and urgent interventions [19]. Although a significant benefit was observed with dual therapy in PUB patients with active bleeding or nonbleeding visible vessels, no stratified analysis based on the Forrest classification (such as Forrest Ib and Forrest IIb) was performed. Forrest Ia and IIa mainly represent arterial bleeding, whereas Forrest Ib mainly represents nonarterial bleeding. Moreover, whether dual therapy has a significant benefit for PUB patients in different Forrest classifications is not clear [20]. Therefore, this study aimed to compare hemostatic efficacy between epinephrine monotherapy and dual therapies by using a propensity score (PS) analysis, and a stratified analysis was performed based on different Forrest classifications.

## Methods and materials

### Patients and study design

This study was a single-center, retrospective, propensity-matched study design. An endoscopy database and clinical records from the First Affiliated Hospital of Nanchang University, Nanchang, China, were retrospectively reviewed. Between March 2014 and June 2022, a total of 936 patients with peptic ulcer bleeding underwent endoscopic epinephrine injections or epinephrine injections combined with a second therapy for hemostasis, which included sclerosant injection, titanium clip hemostasis, and thermal coagulation. Patients meeting the following criteria were excluded from the analysis: (1) greater than two types of hemostasis methods used; (2) patients diagnosed with other possible reasons for bleeding, such as anastomotic ulcers, Dieulafoy lesions, or malignant lesions.; (3) patients with Forrest Ia peptic ulcers, which rarely underwent endoscopic epinephrine injections alone, as well as Forrest IIc and III peptic ulcers, which were not necessary for endoscopy intervention for hemostasis; and (4) patients with incomplete demographic data. Finally, a total of 605 patients were enrolled in the study. We collected the patient’s medical information, including demographic information, physical examinations, laboratory findings, endoscopic findings, Glasgow Blatchford score, Rockall score, AIMS65 score, pharmacological therapy after endoscopic hemostasis, and clinical outcomes [21–23]. The study was approved by the Human Ethics Committee of The First Affiliated Hospital of Nanchang University. All of the patients provided written informed consent for the endoscopic procedure.

### Endoscopic evaluation and medication

All of the emergency endoscopic treatments were performed by experienced deputy directors or chief physicians within 24 h. Endoscopists were familiar with the indications, efficacy, and limitations of the tools and techniques that were currently available for endoscopic hemostasis [11, 13]. For patients with gastrointestinal bleeding who use low-dose antiplatelet drugs as monotherapy for primary cardiovascular prophylaxis, the use of antiplatelet drugs should be temporarily discontinued. For patients with gastrointestinal bleeding who received antiplatelet therapy for secondary prevention of cardiovascular disease, a single antiplatelet drug should not be interrupted, while in those on dual antiplatelet therapy, one of the agents should be temporarily discontinued and re-administered as soon as possible [14]. These individuals were skilled in applying endoscopic hemostasis therapy, and all of them had more than 5 years of endoscopic experience. In this study, we chose patients who underwent endoscopic epinephrine injections alone or epinephrine injections combined with a second method between March 2014 and June 2022 for enrollment. Diluted epinephrine (1:10,000 dilution, equivalent to 100 mcg/mL) was injected at or near the bleeding site [24]. For cases of difficult endoscopic hemostasis, appropriate hemostatic methods should be used for initial hemostasis, followed by timely interventional or surgical procedures. All of the enrolled patients ultimately achieved technical hemostasis. The bleeding status under endoscopy was classified based on the modified Forrest classification. After endoscopy, patients subsequently received high-dose intravenous PPIs (the HD-IVP group, an 80 mg bolus injection followed by a continuous infusion of 8 mg per hour for 72 h) or standard-dose intravenous PPIs (the SD-IVP group, 40 mg infusion twice daily for 72 h), including esomeprazole or pantoprazole. Afterwards, 40 mg PPI was taken orally once daily for 30 days after short-term (72 h) high-dose intravenous PPI therapy in the hospital. All of the patients were followed up for at least 30 days.

### Definition

Rebleeding was defined as recurrent hematemesis, melena, anemia, or vital hemodynamic instability with a decrease in hemoglobin by at least 2 g/dL after a successful initial endoscopic treatment within 30 days, and fresh blood could be observed in the stomach or duodenum during the second-look endoscopic observation [13, 25]. Patients who underwent a second endoscopic therapy for hemostasis within 30 days were also regarded as experiencing rebleeding. Shock was defined as a shock index (pulse rate/systolic blood pressure) > 1.0 or systolic blood pressure < 90 mmHg.

### Outcomes and statistical analysis

Outcomes and statistical analyses were performed with IBM SPSS software version 25.0 for Windows (SPSS Inc., Chicago, IL, USA) and R statistical software 4.1.0 (www.r-project.org). A two-tailed P value < 0.05 was considered to be statistically significant. For normally distributed data, continuous variables are presented as the mean ± standard deviation (SD) and were analyzed by using a Student’s t test. For abnormally distributed data, continuous variables were expressed as the median and interquartile range. The Mann–Whitney U test was performed to analyze the data. Categorical variables were expressed as proportions, and the χ2 test or Fisher’s exact test was used to analyze the data, as appropriate. To control and reduce the selection bias and other potential confounders in the retrospective studies, a propensity score (PS) analysis was performed as a nonrandomized sensitivity analysis. PS was estimated by using a multivariable logistic regression model with the following covariates: sex, age, ulcer size, ulcer location, Forrest classification, medication history (use of nonsteroidal anti-inflammatory drugs, use of anticoagulants, and use of antiplatelets), PUB history, coexisting diseases (hypertension and diabetes mellitus), Rockall score, AIMS65 score, GBS, PPI use, and heart rate, among other factors. The epinephrine injection monotherapy group was matched to the combined therapy group in a 1:1 ratio by using the nearest neighbor method with a caliper width of 0.1. After matching, all of the baseline characteristics were balanced (P > 0.05) between the two groups.

Based on the different Forrest classifications, the recurrent bleeding rates were calculated in the monotherapy and combination therapy groups by Days 3, 7, 14, and 30. The χ2 test or Fisher’s exact test was used to compare the monotherapy and combination therapy groups, as appropriate. Furthermore, the Kaplan‒Meier method was used to analyze the rebleeding rate within 30 days.

## Results

### Baseline characteristics of patients

**Fig. 1:**
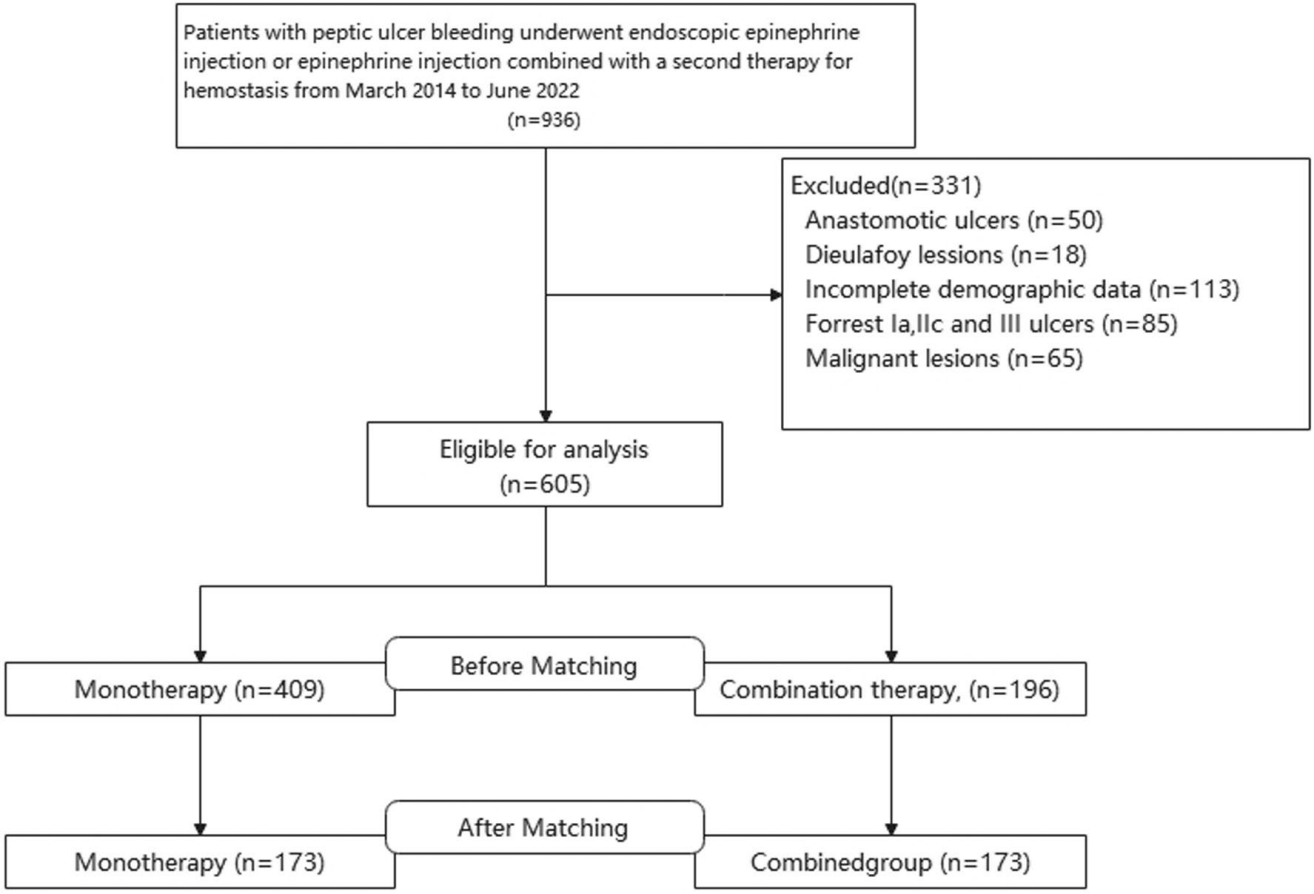
Flowchart of patients included in this study

Between March 2014 and June 2022, a total of 936 patients with peptic ulcer bleeding were screened, and 605 patients who met the inclusion criteria were enrolled, with 409 patients in the monotherapy group and 196 patients in the combination therapy group (Fig. 1). Tables 1, 2, 3 present the baseline characteristics of the enrolled patients. Of the 605 enrolled patients, the median age was 51 (IQR 37–63) years, and the majority (83.0%) were male. In addition, the most common site of peptic ulcer bleeding was the duodenum (66.1%), followed by the stomach (33.9%), and at least 9.3% of patients had large ulcers (> 20 mm). Only 35 (5.8%) patients had hypotension (systolic blood pressure < 90 mm Hg), but up to 19% of the patients had hemorrhagic shock (shock index > 1), which was mostly observed (71.4%) in the monotherapy group. Concerning the laboratory results, the median hemoglobin (HB) level at admission was 87 (IQR 70–110) g/L, and the albumin (ALB) measurement was 36 (31–40) g/L. Before PS matching (PSM), there were differences (P < 0.05) in many of the baseline variables between the two groups, such as sex, PUB history, and hemoglobin. After PSM, there were three deaths in the monotherapy group, including two patients who died due to hemorrhagic shock caused by gastrointestinal bleeding, and another patient who died from multiple organ failure caused by acute severe pancreatitis. A total of 173 patients receiving monotherapy were matched with 173 patients receiving combination therapy after PSM. There were no significant differences in the baseline variables between the two groups (Tables 1, 2, 3).

**Table 1:** Baseline characteristics before and after propensity score matching

Characteristic	Total	Before matching		P	After matching		P
		MT group (n = 409)	CT group (n = 196)		MT group (n = 173)	CT group (n = 173)	
Median age, median (IQR)	51 (37–63)	50 (37–61)	51 (35–65)	0.502	51 (39–62)	50 (34–64)	0.696
Sex: male, no. (%)	502 (83.0)	348 (85.1)	154 (78.6)	0.046	139 (80.3)	142 (82.1)	0.680
Alcohol use, no. (%)	110 (18.2)	74 (18.1)	36 (18.4)	0.935	24 (13.9)	33 (19.1)	0.192
Smokers, no. (%)	171 (28.3)	118 (28.9)	53 (27.0)	0.644	38 (22.0)	50 (28.9)	0.139
Medication history							
Use of antiplatelets, no. (%)	29 (4.8)	22 (5.4)	7 (3.6)	0.330	9 (5.2)	7 (4.0)	0.609
Use of anticoagulants, no. (%)	8 (1.3)	6 (1.5)	2 (1.0)	1	5 (2.9)	2 (1.2)	0.448
Use of NSAIDs, no. (%)	29 (4.8)	19 (4.6)	10 (5.1)	0.806	8 (4.6)	9 (5.2)	0.804
Coexisting diseases, no. (%)							
Hypertension	140 (23.1)	105 (25.7)	35 (17.9)	0.033	33 (19.1)	34 (19.7)	0.892
Diabetes mellitus	54 (8.9)	31 (7.6)	23 (11.7)	0.093	16 (9.2)	16 (9.2)	1
PUB history, no. (%)	138 (22.8)	108 (26.4)	30 (15.3)	0.002	34 (19.7)	28 (16.2)	0.400
Systolic blood pressure < 90, no. (%)	35 (5.8)	25 (6.1)	10 (5.1)	0.618	4 (2.3)	9 (5.2)	0.157
Heart rate > 100 beats/min, no. (%)	111 (18.3)	67 (16.4)	44 (22.4)	0.071	41 (23.7)	38 (22.0)	0.701
Heart rate	85 (74–96)	85 (74–96)	87 (76–100)	0.134	85 (74–100)	88 (75–99)	0.826
Bleeding to shock, no. (%)	115 (19.0)	70 (17.1)	45 (23.0)	0.086	34 (19.7)	39 (22.5)	0.510
GBS, median (IQR)	10 (7–11)	10 (7–11)	10 (7–12)	0.479	10 (7–12)	10 (7–12)	0.797
Rockall score, median (IQR)	4 (3–5)	4 (3–5)	4 (3–5)	0.301	4 (3–5)	4 (3–5)	0.705
AIMS65 score, median (IQR)	0 (0–1)	0 (0–1)	0 (0–1)	0.995	0 (0–1)	0 (0–1)	0.391

*MT* epinephrine injection monotherapy, *CT* epinephrine injection combined with a second therapy, *GBS* Glasgow-Blatchford score, *IQR* interquartile range, *NSAID* nonsteroidal anti-inflammatory drug, *PUB* peptic ulcer bleeding

**Table 2:** Laboratory findings before and after propensity score matching

Characteristic	Total	Before matching		P	After matching		P
		MT group (n = 409)	CT group (n = 196)		MT group (n = 173)	CT group (n = 173)	
Low HB level < 100 g/L, no. (%)	391 (64.6)	258 (63.1)	133 (67.9)	0.250	114 (65.9)	115 (66.5)	0.910
Hemoglobin level on admission, g/L, median (IQR)	87 (70–110)	88 (72–112)	83 (66–107)	0.036	86 (71–107)	84 (66–107)	0.514
White cell count, × 109/L, median (IQR)	9 (7–12)	9 (7–12)	9 (6–11)	0.649	9 (7–12)	9 (7–11)	0.691
PLT, < 100 × 10^9^/L, no. (%)	64 (10.6)	39 (9.5)	25 (12.8)	0.228	16 (9.2)	23 (13.3)	0.234
Platelet, × 109/L, median (IQR)	187 (141–235)	189 (146–238)	179 (130–227)	0.202	189 (143–242)	181 (130–229)	0.371
Blood urea nitrogen, mmol/L, median (IQR)	10 (7–13)	10 (7–13)	10 (7–14)	0.487	10 (6–13)	10 (7–13)	0.434
Creatinine, μmol/L, median (IQR)	73 (61–87)	74 (62–88)	72 (60–86)	0.209	70 (61–85)	72 (61–86)	0.967
ALB, < 30 g/L, no. (%)	131 (21.7)	80 (19.6)	51 (26.0)	0.071	37 (21.4)	43 (24.9)	0.444
Albumin g/L, median (IQR)	36 (31–40)	36 (31–40)	35 (29–40)	0.199	36 (31–40)	36 (30–40)	0.564
Prothrombin time, s, median (IQR)	12 (11–13)	12 (11–13)	12 (11–13)	0.132	12 (11–13)	12 (11–13)	0.806
APTT, median (IQR	25 (22–28)	24 (22–28)	25 (22–30)	0.198	24 (23–27)	25 (22–29)	0.342
INR > 1.5, no. (%)	15 (2.5)	9 (2.2)	6 (3.1)	0.579	4 (2.3)	6 (3.5)	0.521
INR	1.03 (0.97–1.11)	1.03 (0.97–1.11)	1.03 (0.97–1.12)	0.456	1.03 (0.99–1.10)	1.03 (0.96–1.12)	0.549

*ALB* albumin, *APTT* activated partial thromboplastin time, *HB* hemoglobin, *MT* epinephrine injection monotherapy, *CT* epinephrine injection combined with a second therapy, *INR* international normalized ratio, *IQR* interquartile range

**Table 3:** Endoscopic findings and pharmacological therapy before and after propensity score matching

Characteristic	Total	Before matching		P	After matching		P
		MT group (n = 409)	CT group (n = 196)		MT group (n = 173)	CT group (n = 173)	
Ulcer size > 2 cm, no. (%)	56 (9.3)	39 (9.5)	17 (8.7)	0.732	18 (10.4)	17 (9.8)	0.858
Ulcer size, mm, median (IQR)	0.8 (0.5–1)	0.8 (0.5–1.2)	0.8 (0.5–1)	0.021	0.8 (0.5–1)	0.8 (0.5–1)	0.500
Ulcer location, no. (%)				0.001			0.913
Duodenum	400 (66.1)	293 (71.6)	107 (54.6)		99 (57.2)	100 (57.8)	
Stomach	205 (33.9)	116 (28.4)	89 (45.4)		74 (42.8)	73 (42.2)	
Stigmata of hemorrhage, no. (%)				0.001			0.167
Forrest Ib	195 (32.2)	115 (28.1)	80 (40.8)		74 (42.8)	68 (39.3)	
Forrest IIa	225 (37.2)	141 (34.5)	84 (42.9)		57 (32.9)	73 (42.2)	
Forrest IIb	185 (30.6)	153 (37.4)	32 (16.3)		42 (24.3)	32 (18.5)	
Intravenous PPI infusion after endoscopic hemostasis, no. (%)				0.625			0.542
Esomeprazole	449 (74.2)	306 (74.8)	143 (73.0)		130 (75.1)	125 (72.3)	
Pantoprazole	156 (25.8)	103 (25.2)	53 (27.0)		43 (24.9)	48 (27.7)	
PPI therapy, no. (%)				0.016			1
SD-IVP	141 (23.3)	107 (26.2)	34 (17.3)		33 (19.1)	33 (19.1)	
HD-IVP	464 (76.7)	302 (73.8)	162 (82.7)		140 (80.9)	140 (80.9)	

*MT* epinephrine injection monotherapy, *CT* epinephrine injection combined with a second therapy, *SD-IVP* standard-dose intravenous proton pump inhibitor, 40 mg infusion twice daily for a period of 72 h, *HD-IVP* high-dose intravenous proton pump inhibitor, an 80 mg bolus injection followed by a continuous infusion of 8 mg per hour for a period of 72 h, *IQR* interquartile range, *PPI* proton pump inhibitor

### Outcome measures after endoscopic hemostasis

Compared with the combination therapy group, the monotherapy group showed significantly higher rates of recurrent bleeding by Days 3, 7, 14, and 30 (12.1%, 16.8%, 17.9%, and 17.9%, respectively, vs. 5.8%, 8.1%, 9.2%, and 9.8%, respectively; P < 0.05) after PSM (Table 4). After PSM, the monotherapy and combination therapy groups did not significantly differ in secondary endoscopy, mortality, interventional procedures, or length of hospital stay.

**Table 4:** Outcome measures after endoscopic hemostasis before and after propensity score matching

Characteristic	Before matching		P	After matching		P
	MT group (n = 409)	CT group (n = 196)		MT group (n = 173)	CT group (n = 173)	
Recurrent bleeding, no. (%)						
By day 3	39 (9.5)	11 (5.6)	0.101	21 (12.1)	10 (5.8)	0.038
By day 7	52 (12.7)	15 (7.7)	0.063	29 (16.8)	14 (8.1)	0.015
By day 14	55 (13.4)	17 (8.7)	0.09	31 (17.9)	16 (9.2)	0.019
By day 30	59 (14.4)	19 (9.7)	0.104	31 (17.9)	17 (9.8)	0.029
Outcome, no. (%)						
Secondary endoscopy	34 (8.3)	13 (6.6)	0.520	16 (9.2)	11 (6.4)	0.672
Mortality	8 (2.0)	0 (0)	0.059	3 (1.7)	0 (0)	0.248
Interventional surgery	10 (2.4)	3 (1.5)	0.563	6 (3.5)	3 (1.7)	0.199
Surgery	7 (1.7)	2 (1.0)	0.725	6 (3.5)	2 (1.2)	0.685
Median hospital stay > 7 d, no. (%)	113 (27.6)	70 (35.7)	0.043	56 (32.4)	58 (33.5)	0.819
Hospitalization stay, range	1–71	0–63		1–53	0–54	

*MT* epinephrine injection monotherapy, *CT* epinephrine injection combined with a second therapy

### Recurrent bleeding rates by Days 3, 7, 14, and 30 among different Forrest classifications

**Fig. 2:**
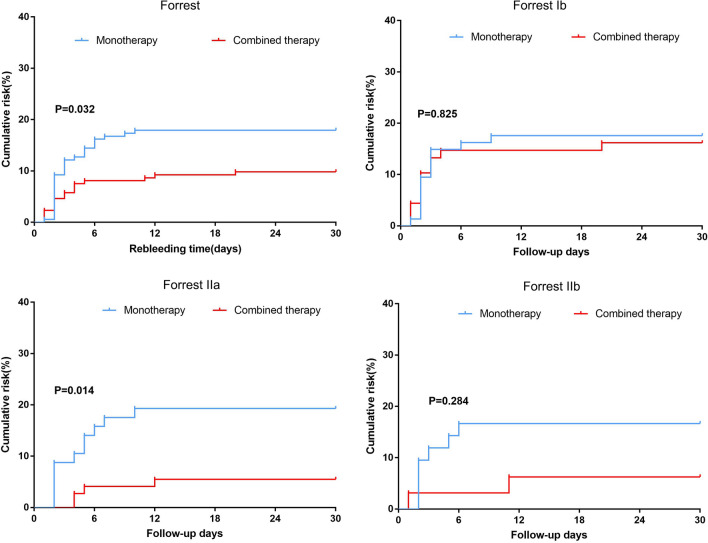
Cumulative recurrent bleeding rates within 30 days among different Forrest classifications. Forrest; Forrest Ib; Forrest IIa; Forrest IIb

Tables 5, 6 present the recurrent bleeding rates by Days 3, 7, 14, and 30 among different Forrest classifications in the MT and CT groups before and after PSM. Although the rebleeding rate was more frequently observed in the MT group than in the CT group, there was no significant difference in PUB patients with oozing blood (FIb) or adherent clots (FIIb) between the MT group and CT group (both before and after PSM). For PUB patients with oozing bleeding (FIb), the recurrent bleeding rates by Days 3, 7, 14, and 30 after PSM were 14.9%, 16.2%, 17.6%, and 17.6% in the MT group, respectively, and rates were 13.2%, 14.7%, 14.7%, and 16.2% in the CT group, respectively, with no significant differences observed between the 2 groups by Days 3, 7, 14, and 30 (P = 0.78, P = 0.804, P = 0.644, and P = 0.825, respectively). For PUB patients with nonbleeding visible vessels (FIIa), the recurrent bleeding rates by Days 3, 7, 14, and 30 after PSM were 8.8%, 17.5%, 19.3%, and 19.3% in the MT group, respectively, and rates were 0%, 4.1%, 5.5%, and 5.5% in the CT group, respectively, with significant differences observed between the 2 groups (P = 0.015, P = 0.011, P = 0.014, and P = 0.014, respectively). Figure 2 shows the patient’s cumulative recurrent bleeding rates within 30 days among different Forrest classifications before and after PSM. The Kaplan‒Meier analysis demonstrated a significant difference between the monotherapy group and the combination therapy group during the 30 day follow-up period (P = 0.032) after PSM. Likewise, PUB patients with oozing blood (FIb) and adherent clots (FIIb) had similar outcomes (P = 0.825 and P = 0.284, respectively). In contrast, ulcers with nonbleeding visible vessels (FIIa) differed between the monotherapy group and the combination therapy group (P = 0.014).

**Table 5:** Recurrent bleeding rates by days 3, 7, 14, and 30 among different Forrest classifications before propensity score matching

Stigmata of hemorrhage	By day 3		P	By day 7		P	By day 14			By day 30		P
	MT group n/N(%)	CT group n/N(%)		MT group n/N(%)	CT group n/N(%)		MT group n/N(%)	CT group n/N(%)	P	MT group n/N(%)	CT group n/N(%)	
Forrest Ib (n = 195)	17/115 (14.8)	9/80 (11.3)	0.475	21/115 (18.3)	10/80 (12.5)	0.279	23/115 (20.0)	10/80 (12.5)	0.169	24/115 (20.9)	12/80 (15.0)	0.299
Forrest IIa (n = 225)	12/141 (8.5)	1/84 (1.2)	0.035	18/141 (12.8)	4/84 (4.8)	0.051	19/141 (13.5)	5/84 (6.0)	0.077	22/141 (15.6)	5/84 (6.0)	0.031
Forrest IIb (n = 185)	10/153 (6.5)	1/32 (3.1)	0.693	13/153 (8.5)	1/32 (3.1)	0.47	13/153 (8.5)	2/32 (6.3)	1	13/153 (8.5)	2/32 (6.3)	1

*MT* epinephrine injection monotherapy, *CT* epinephrine injection combined with a second therapy

**Table 6:** Recurrent bleeding rates by days 3, 7, 14, and 30 among different Forrest classifications after propensity score matching

Stigmata of hemorrhage	By day 3		P	By day 7		P	By day 14		P	By day 30		P
	MT group n/N(%)	CT group n/N(%)		MT group n/N(%)	CT group n/N(%)		MT group n/N(%)	CT group n/N(%)		MT group n/N(%)	CT group n/N(%)	
Forrest Ib (n = 142)	11/74 (14.9)	9/68 (13.2)	0.78	12/74 (16.2)	10/68 (14.7)	0.804	13/74 (17.6)	10/68 (14.7)	0.644	13/74 (17.6)	11/68 (16.2)	0.825
Forrest IIa (n = 130)	5/57 (8.8)	0/73 (0)	0.015	10/57 (17.5)	3/73 (4.1)	0.011	11/57 (19.3)	4/73 (5.5)	0.014	11/57 (19.3)	4/73 (5.5)	0.014
Forrest IIb (n = 74)	5/42 (11.9)	1/32 (3.1)	0.226	7/42 (16.7)	1/32 (3.1)	0.127	7/42 (16.7)	2/32 (6.3)	0.284	7/42 (16.7)	2/32 (6.3)	0.284

*MT* epinephrine injection monotherapy, *CT* epinephrine injection combined with a second therapy

## Discussion

Peptic ulcer bleeding has a high incidence of rebleeding within 30 days after endoscopic hemostasis and can lead to severe complications or even death. Moreover, the high rebleeding rate can impose a serious financial burden on patients and can seriously affect their quality of life [6, 13, 25]. As the Forrest classification provides prognostic information about the risks of rebleeding, as well as the need for therapeutic interventions and death, it is recommended for the stratification of patients with bleeding ulcers and for guiding management decisions, including endoscopic and pharmacological treatments. Therefore, the choice of treatment modality for different Forrest classifications is essential. Most guidelines and clinical trials strongly recommend that for peptic ulcer bleeding with high-risk stigmata (active bleeding or visible vessels), a second hemostatic modality (such as thermal, mechanical, or sclerotherapy injection modalities) combined with epinephrine injections can significantly reduce rebleeding rates and provide a favorable prognosis [9–18, 26, 27]. In fact, epinephrine injection monotherapy also has many advantages, such as high efficacy, ease of performance, fewer technical requirements, and low costs. Therefore, it is widely used in clinical practice. However, there have been few studies on which treatment modality is used for different classifications, and confounding factors exist. Compared with epinephrine injection monotherapy, whether dual therapy has a significant benefit for PUB patients in different Forrest classifications is unclear. A previous study demonstrated a significant benefit with dual therapy in PUB patients with active bleeding and in those patients with nonbleeding visible vessels compared with epinephrine injection monotherapy [9–18, 26]. Theoretically, Forrest Ia and IIa mainly represent arterial bleeding, and the hemostatic efficacy of dual therapy is better than that of epinephrine monotherapy in these types of patients, whereas Forrest Ib mainly represents nonarterial bleeding; therefore, the hemostatic efficacy of epinephrine injection therapy is potentially comparable to that of dual therapy [27]. In our study, the results showed that the hemostatic efficacy of epinephrine injection monotherapy is inferior to that of dual therapy in PUB patients after propensity matching; however, it has similar hemostatic efficacy to dual therapy in PUB patients with Forrest Ib ulcers via a stratified analysis.

Previous studies have demonstrated a significant benefit of epinephrine injections plus the second modality for further bleeding, surgery, and urgent interventions compared with monotherapy [9–18, 26]. In our study, we also analyzed the hemostatic effects of epinephrine injection monotherapy and combination therapy after propensity matching from a detailed database of patients with bleeding peptic ulcers. After using the strict matching method for PSM, which included all of the possible risk-related baseline variables for matching, high-risk patients in the 2 treatment groups were similar, with no significant differences observed in the baseline variables, including the Forrest classification, hemoglobin, blood pressure, and ulcer size. Thus, the two treatment groups were suitable for comparing the efficacy of the two treatments. We also obtained a significant benefit of epinephrine injections plus the second modality for further bleeding; however, there was no significant difference in surgery and emergency intervention in our study, which may be due to the small sample size.

Although a significant benefit of dual therapy for further bleeding was observed in our study, the stratified analysis showed similar results only in FIIa PUB patients, with rebleeding rates of 8.8%, 17.5%, 19.3%, and 19.3% in the monotherapy group by Days 3, 7, 14, and 30, respectively, and rates of 0%, 4.1%, 5.5%, and 5.5% in the combination therapy group, respectively (P < 0.05). Furthermore, monotherapy and dual therapy showed similar hemostatic efficacy in PUB patients with oozing blood (FIb) ulcers, which mainly represent nonarterial bleeding (13/74, 17.6% vs. 11/68, 16.2%, respectively, by Day 30, P = 0.825). Therefore, epinephrine injection monotherapy is also a good choice for PUB patients with oozing blood (FIb) ulcers.

The proportion of arterial bleeding is generally high in PUB patients with FIIb ulcers and higher than in PUB patients with FIb ulcers. Theoretically, a higher percentage of arterial bleeding corresponded to a worsened hemostatic efficacy of monotherapy. Although there was no significant difference in the rebleeding rate of the PUB patients with adherent clot (FIIb) ulcers between the monotherapy group and the combination therapy group both before and after PSM in our study, rebleeding was more frequently observed in the monotherapy group than in the combination therapy group. The small sample size may be the main reason for this result. In addition, the blood clot coverage made it impossible to determine whether the ulcer represented arterial bleeding, and the proportion of arterial bleeding was not clear, which may have affected the results. Therefore, monotherapy may not be a good choice for PUB patients with FIIb ulcers.

This study had several advantages. First, the strict matching method (PSM) was used with all of the possible risk-related baseline variables, which made the two groups suitable for comparing the efficacy of the two treatments. Second, the Forrest classification was adopted for a stratified analysis in this study, which reflects the different bleeding mechanisms. However, there were several limitations to this study. First, this was a single-center retrospective study, which may introduce a selection bias due to the nature of the retrospective studies. Second, endoscopic treatment is performed by different levels of endoscopists and may have subtle differences in prognosis. Finally, the results may be biased due to the small sample size of the PUB patients with adherent blood clot (FIIb) ulcers. Further multicenter and large-sample prospective clinical trials are necessary to validate future findings.

In conclusion, combined therapy has higher hemostatic efficacy than epinephrine injection monotherapy for PUB patients with visible blood vessel (FIIa) ulcers. However, epinephrine injection monotherapy is equally as effective as combined therapy for PUB patients with oozing blood (FIb) ulcers.

## Data Availability

The datasets used and/or analysed during the current study are available from the corresponding author on reasonable request. The data that support the findings of this study are available from the The First Affiliated Hospital of Nanchang University, but restrictions apply to the availability of these data, which were used under license for the current study, and so are not publicly available. Data are however available from the authors upon reasonable request and with permission of The First Affiliated Hospital of Nanchang University.

## Abbreviations

PUB: Peptic ulcer bleeding
PPIs: Proton pump inhibitors
APC: Argon plasma coagulation
HB: Hemoglobin
WBC: White blood cell count
PLT: Platelet
BUN: Blood urea nitrogen
Cr: Creatinine
ALB: Albumin
PT: Prothrombin time
APTT: Activated partial thromboplastin time
IQR: Interquartile range
INR: International normalized ratio
MT: Epinephrine injection monotherapy
CT: Epinephrine injection combined with a second therapy
SD: Standard deviation
SD-IVP: Standard-dose intravenous proton pump inhibitor, 40 mg infusion twice daily for a period of 72 h
HD-IVP: High-dose intravenous proton pump inhibitor, an 80 mg bolus injection followed by a continuous infusion of 8 mg per hour for a period of 72 h
